# Four Years Since COVID-19 Day Zero: A Time to Evaluate Past and Future Pandemic Control Policies and Practices in Sub-Saharan Africa?

**DOI:** 10.2147/RMHP.S449701

**Published:** 2024-03-05

**Authors:** Obinna O Oleribe, Andrew W Taylor-Robinson, Okey C Nwanyanwu, Marsha Y Morgan, Simon D Taylor-Robinson

**Affiliations:** 1 Office of the Director-General, Nigerian Institute for Medical Research, Lagos, Nigeria; 2 Best Health Consult Limited Liability Company, Orange, CA, USA; 3 College of Health Sciences, VinUniversity, Hanoi, Vietnam; 4 Center for Global Health, Perelman School of Medicine, University of Pennsylvania, Philadelphia, PA, USA; 5 Fast Forward Africa Limited Liability Company, Austin, TX, USA; 6 Division of Medicine, Royal Free Campus, University College London, London, UK; 7 Departments of Medicine & Public Health, Busitema University, Mbale, Uganda; 8 Department of Surgery and Cancer, Imperial College London, St Mary’s Hospital Campus, London, UK

**Keywords:** sub-Saharan Africa, COVID-19, health inequality, non-pharmaceutical interventions, pandemic, vaccination

## Abstract

Four years after the first case of COVID-19, the world is still determining how best to prevent and control the long-term effects of SARS-CoV-2 infection. Non-pharmaceutical interventions (NPIs) were employed at the start of the pandemic as the only available options, prior to effective vaccines and antiviral agents. The World Health Organization recommended dual vaccination for 70% worldwide as the threshold for a return to “normal” community life. Immunization rates needed to increase in all global regions, irrespective of socioeconomic status, necessitating more equitable access. During the pandemic, wealthier countries hoarded vaccine supplies even when their citizens were immunized. This highlights the already enormous difficulties in healthcare provision faced by low-income sub-Saharan African countries, which remain at risk as industrialized nations have progressed to a post-pandemic era. Thus, in addition to redoubling vaccination efforts public health policymakers should consider ongoing and future use of NPIs. In this narrative account, we advocate that various NPI practices should not be shelved; rather, more research is needed to evaluate their impact in parallel with booster vaccination. This especially applies to so-called “long COVID”. Lessons learned from implementing best practices in resource-limited settings should be incorporated into preparedness guidelines for future infectious disease outbreaks.

## Introduction

Much has been written on the global pandemic caused by severe acute respiratory syndrome coronavirus (SARS-CoV)-2 infection.^1–4^ SARS-CoV-2, in common with two other pathogenic human respiratory coronaviruses, SARS-CoV and MERS-CoV, tends to cause symptoms at the severe end of the respiratory disease spectrum.^5^,^6^ By comparison, infections with four other coronaviruses, HKU1, NL63, 229E and OC43, generally produce much milder symptoms, often referred to as the “common cold”.^7^

With the initial emergence of human SARS-CoV-2 infections in December 2019, subsequently termed “coronavirus disease 2019” (COVID-19), the first response of governments around the world was to try to prevent and control viral transmission through public health interventions.^8^ Efforts escalated as the virus spread from China to Western Europe and North America in January 2020, with African cases following shortly afterwards.^9^ When the World Health Organization (WHO) declared COVID-19 to be a “public health emergency of international concern” on January 30, 2020,^10^ and then on March 11, 2020, confirmed that the world was facing a pandemic,^11^ the global strategy focused first on disease prevention and later on accelerated development of vaccines and antiviral agents.^6^ In the interim, most countries adopted hastily arranged interventions, whereby national borders were closed, air travel was suspended, schools and offices were shut, and remote working and learning were promoted.^12^,^13^ The introduction of personal protective measures meant that people across sub-Saharan Africa and other parts of the world were asked to wear masks, wash their hands frequently or to use hand sanitizers, maintain social distancing, stay at home wherever possible, avoid hand-to-face contact and minimize socialization with others, including shaking hands and other more intimate greetings.^14–19^

In addition, screening policies were implemented in Africa using newly developed viral antigen testing; exposed individuals were required to quarantine and those testing positive were asked to self-isolate if it was clinically safe to do so.^7^,^15^,^17^ Those who developed more severe symptoms were either treated in isolation centres or referred to public and private facilities for care, if such facilities were available.^14^ The most seriously ill patients were moved to intensive care units (ICUs), where such facilities existed. As the numbers of admitted cases increased, hospitals began to run out of beds, in response to which, in some countries, temporary housing was made available, such as in hastily but adequately equipped sporting venues.^20^ In some instances, hospital and ICU capacity was exceeded, meaning that difficult choices had to be made about which patients would or would not be further supported.^20^

In other parts of the world, by comparison, different strategies were adopted which took advantage of locally available resources. One interesting example of a COVID-19 non-pharmacological intervention (NPI) comes from Thailand. There, especially during the Delta variant wave, the government implemented various types of temporary facilities, including field hospitals and “hospitel” (a hybrid hospital and hotel with essential medical equipment, such as radiography), as well as enforcing home isolation (with telemedicine consultation). Some premises, such as schools, temples, sports venues and auditoriums, were used as “state quarantine” or field hospitals.^21^

In Africa, schools and businesses were closed, with people asked or even forced by “lockdown” measures to stay indoors, mostly either alone or with close family members only.^22^ Persons considered to be particularly vulnerable, for example, the elderly, the immune compromised and those with cancer were required to shield for weeks or months on end, often to the detriment of their mental and physical health.^23^ However, whether these NPIs had a significant benefit on case growth is the subject of ongoing research.^24^

Healthcare facilities and personnel were so overwhelmed with caring for patients with COVID-19 that many routine services, and even cancer screening and treatment, were severely reduced or stopped altogether.^15^ The fear of contracting COVID-19 meant that even when healthcare facilities were available, many people chose not to access them; some mistakenly believed that not “bothering doctors” was the right thing to do when services were so stretched.^1^,^2^ Many were afraid of the social stigma of doing so, while others were afraid of close contact with healthcare workers who had been treating the sick.^25^ Such hesitancy to visit care centres resulted in increased mortality, morbidity and complications from both infectious and metabolic diseases, such as malaria, HIV/AIDS, diabetes and hypertension, particularly in sub-Saharan Africa.^26^ As hospitals became overwhelmed, healthcare workers became victims of pandemic-related burnout, fatigue and stress.^27^ Not unexpectedly, many front-line workers succumbed to the virus; many resigned because of the continued pressure and anxiety engendered by looking after very sick patients, many of whom died, whilst others took long leave to look after their families and to provide home schooling.^27^

In many countries people were not allowed to visit their very sick relatives in hospital and care homes to provide emotional support, even at end of life.^27^ Patients who died were not given otherwise normal or culturally appropriate burials.^28^ Families were not permitted to attend the funerals of their relatives, in accordance with social distancing policy, in some cases even after vaccination.^28^ Some were buried in mass graves without next-of-kin involvement.^28^ Other important life events, such as coming of age celebrations, milestone birthdays and weddings were either banned or celebrated with a minimum of attendees.^28^

## Advent of Vaccines and Antivirals

The early promotion of drugs such as ivermectin, chloroquine and hydroxychloroquine, which were then conclusively shown not to confer benefit, negatively affected public perception of ethically approved, scientifically proven strategies like mass vaccination programs.^29^ It could be argued that as a result the general public lost trust in medical, scientific and governmental authorities.^30^ Late in 2020, COVID-19 vaccines first became available, initially in Europe; those manufactured by Pfizer-BioNTech and Moderna, were introduced under Emergency Use Authorization.^31^ With the introduction of vaccines came vaccine hesitancy, which to a degree still continues.^32^,^33^ Furthermore, vaccines were initially almost exclusively available to wealthy, industrialized nations while low- and middle-income countries struggled with poor supply chains and delivery infrastructures, or else were offered surplus stocks with very short expiry dates, particularly in sub-Saharan Africa.^34^,^35^ Even when vaccines were available, difficulties encountered in cold chain systems, particularly for mRNA vaccines, limited supply to rural areas.^36^

The combination of uncoordinated public health policies and community non-compliance led to poor rates of vaccine uptake in some parts of the world.^37^ This resulted in delays in achieving threshold levels of mass vaccination, during which time the globally predominant variant of SARS-CoV-2 mutated several times, possibly contributing to the global failure to achieve herd immunity.^38^ The successive emergence of these antigenically distinct viral mutations led to multiple epidemic spikes, with subsequent increases in mortality and morbidity that perhaps could have been avoided if more people had been vaccinated.^39^

## From Pandemic to Post-Pandemic

On January 24, 2022, Hans Kluge, the WHO Regional Director for Europe announced that “The Omicron variant has moved the Covid-19 pandemic into a new phase and could bring it to an end in Europe”.^40^ Anthony Fauci, the Director of the US National Institute of Allergy and Infectious Diseases and Chief Medical Advisor to the US President, expressed similar optimism. He said that if the recent fall in case numbers in areas like the US’s North-East continued, “I believe that you will start to see a turnaround throughout the entire country”.^41^ Of particular interest, the WHO Regional Office in Africa stated that cases of COVID-19 had plummeted in the region and deaths were declining for the first time since the Omicron-dominated fourth wave of the virus reached its peak.^42^

According to official figures compiled by Reuters news agency, to July 15, 2022, at which time daily tracking of cases had ceased but COVID-19 infections were still rising in 72 countries, the pandemic had directly killed 6.78 million people worldwide, with 256,000 confirmed deaths recorded in Africa.^43^ Due to under-reporting, intentionally as a matter of political expediency or simply due to a lack of confirmed cause of death on a vast scale,^44^ the true toll of COVID-19 may never be known, especially in sub-Saharan Africa where public health resources were stretched beyond breaking point.^45^ With the increasing availability of vaccines that can be modified to cope with new COVID-19 variants, Hans Kluge stated that emphasis ought to be on “minimising disruption of hospitals, schools and the economy, and putting huge efforts [into] protecting the vulnerable”, rather than measures to stop transmission. Meanwhile, he urged people to exercise personal responsibility.^40^

## Key Control and Prevention Issues

Thus, as the world seemingly enters a post-pandemic phase with the WHO declaring the global health emergency over in May 2023,^46^ now is the time to address the long-term surveillance, control and prevention of COVID-19, the findings of which will inform our actions towards future emerging (respiratory) pathogens. Several key research questions remain unanswered, including:

1. Is future screening of international travellers and recently exposed individuals epidemiologically and economically justified? While the consensus opinion would be no, Hans Kluge was mindful to say, ”This virus has surprised [us] more than once so, we have to be very careful”.^40^ The WHO is also careful to point out that there is continued COVID-19 infection all around the world, with 6.9 million deaths at the time of writing.^46^ Thus, complacency would be inappropriate and potentially dangerous.
2. Is continued quarantining – mandatory or voluntary – of exposed individuals beneficial to the post-pandemic control of infection in the absence of the emergence of new more pathogenic viral variants? Some African studies that have examined quarantining during the pandemic would suggest not,^47^ but further investigation is required to answer this question definitively in order for coherent post-pandemic strategies to be formulated.
3. Does continued use of masks – mandatory or voluntary – and habitual social distancing help to control spread of infection, or do these measures merely increase the health and economic burden on populations fatigued by the duration of prior control measures?^48^ Again, the published literature is not consistent on this issue, with some African-focused retrospective analyses on pandemic policy implying that these measures may not be helpful over the long-term.^49^ Hence, there needs to be a focus on post-pandemic era strategies so that appropriate guidelines can be developed predicated on a clear evidence base.
4. Why has COVID-19 been the only notifiable disease for which non-infected but “exposed” persons are screened and quarantined, despite both the rate of infection conversion and the rate of mild infection in exposed and infected persons being very low? While this is a matter for renewed investigation, initial mathematical modelling has supported the idea of early contact tracing rather than mass lockdown.^50^ Whether this is an economically viable option in the future remains to be seen, particularly in resource-poor settings.
5. Where is the scientific evidence that any measures other than vaccination, social distancing and possibly the use of face masks work? Although containment measures can be considered in purely virological terms, there are multiple economic, psychosocial and environmental factors to be balanced that were not considered when NPI policies were drawn up rapidly during the pandemic.^51^
6. Which types of face mask are beneficial and to what extent? Given the lack of guidance in many African countries and a supply deficit, particularly to impoverished communities, updated or newly prepared policies should be examined closely for their practicality and effectiveness in the post-pandemic era.^52^

The availability of vaccination and antiviral drugs, although not universal, may allow some NPI practices to be re-evaluated, modified, scaled down or even phased out entirely,^53^ provided that new highly pathogenic viral variants do not emerge.

The most important task remains to ensure that as many people as possible worldwide are at least dually vaccinated, achieving the target of 70% coverage set by the WHO in June 2021.^54^ When the roadmap was first released the intended goal was to increase global population immunity substantially in order to protect as many people as possible from disease, safeguard the functioning of overburdened national healthcare systems, fully restart countries’ economies, restore the public health of societies, and lower the risk posed by new variants of SARS-CoV-2.^55^ The initial strategy aimed to prioritize healthcare workers, the elderly, and high-risk individuals with important co-morbidities, advancing next to all adults, followed by adolescents.^54^

Despite the aspiration to complete this by mid-2022 it has not been achieved, due in large part to confounding of the vaccination campaigns in some African countries by the dual obstacles of poor vaccine supply and high hesitancy rates.^56^ This contrasts with the situation in many developed nations where the latest vaccines are now routinely available, with some individuals receiving four or even five immunizations.^57^ It is, therefore, arguably belatedly, time to rethink vaccine accessibility and distribution, reimagine vaccination strategies, and strive to ensure equity of immunization between nations.^58^ In order to achieve 70% dual vaccine global coverage, it is axiomatic that vaccination rates must rise in low-income economies that are lagging well behind high- and middle-income countries. Moreover, public health messages should focus on increased global coverage of vaccine booster doses.

## Future Public Health Initiatives

Moving forward, based on our collective experiences in Nigeria and Uganda before, during and after the COVID-19 pandemic,^1–3^ we propose that the following measures should be implemented in sub-Saharan Africa:

1. The WHO target of 70% vaccination should be pursued by all governments around the world,^55^ with tailored support offered by external agencies to countries that require help.
2. National biomedical research institutions should conduct blinded retrospective and prospective analytical studies to evaluate the impact of various NPI practices other than hand washing (which is a global public health practice); in the absence of new highly pathogenic viral variants, these should be minimized for administrative authorities that have met the 70% vaccination threshold.^54^
3. The findings of public inquiries into the handling of the pandemic should provide insights into what did and did not “work”, although the results may not be available for some time.
4. Meanwhile, subject matter experts should review global policies and practices, and use their findings to develop regional and national guidelines for control of future pandemics.

## Conclusion

It is four years since the start of COVID-19 pandemic; the current post-pandemic “long COVID” phase is now the right time to review infection control policies and to decide what has and has not been successful. NPIs were useful when SARS-CoV-2 first emerged, prior to the advent of vaccines and therapeutics. It could be argued that now that these pharmaceutical options are available in most regions, the role of NPIs may not be as important and their use should be re-evaluated, based on scientifically robust data. While a case could be made to significantly relax use of NPIs in countries which have achieved the necessary minimum of 70% of the population at least dually vaccinated, in practice, coverage at this level needs to be attained across all continents before decisions on the abandonment of NPIs can be safely made.^55^ This places Africa in the spotlight as the region that is lagging far behind others for vaccination rates. Governments across the continent must contribute and collaborate in a multinational coordinated effort to reduce this inequality in public health provision,^52^,^59^,^60^ in order to achieve the necessary vaccination coverage.

## Approval Statement

The authors confirm that this article does not need institutional approval to be published.
